# Ectopic thymoma presenting as a giant intrathoracic tumor: A case report

**DOI:** 10.1186/1477-7819-9-66

**Published:** 2011-06-28

**Authors:** Masahiro Kitada, Kazuhiro Sato, Yoshinari Matsuda, Satoshi Hayashi, Yoshihiko Tokusashi, Naoyuki Miyokawa, Tadahiro Sasajima

**Affiliations:** 1 Department of Surgery, Asahikawa Medical University, Midorigaoka-Higashi 2-1-1-1, Asahikawa, Hokkaido 078-8510, Japan; 2 Department of Clinical Pathology, Asahikawa Medical University, Midorigaoka-Higashi 2-1-1-1, Asahikawa, Hokkaido 078-8510, Japan

## Abstract

Ectopic thymoma rarely presents as an intrathoracic tumor. We report a case of ectopic thymoma presenting as a giant right intrathoracic tumor that was treated with resection. The patient was a 50-year-old Japanese woman who presented with the chief complaint of chest pain. Detailed examination revealed a solid tumor measuring 15 × 10 × 8 cm in diameter, with a clear border. The Imaging findings suggested a solitary fibrous tumor, and surgery was performed. At surgery, the tumor was found to beadherent to the diaphragm, mediastinal pleura, and lower lobe of the lung, although it could be dissected with relative ease and was removed. Pathological diagnosis indicated a type B1 tumor with no capsular invasion according to the World Health Organization classification, and a diagnosis of Masaoka stage I thymoma was made. No continuity with the normal thymus tissue was seen, and the thymoma was considered to be derived from ectopic thymic tissue in the pleura.

## Background

Thymomas usually manifest in the anterior-superior mediastinum, and ectopic thymomas account for only 4% of all thymomas. Among ectopic thymoma, intrathoracic tumors of pleural origin are rather rare. We report, herein, a patient with a giant intrathoracic tumor that was discovered during a clinical workup to determine the cause of chest pain in the patient. The tumor was diagnosed as a thymoma that was difficult to differentiate from solitary fibrous tumor (SFT) by diagnostic imaging.

## Case

**Figure 1:**
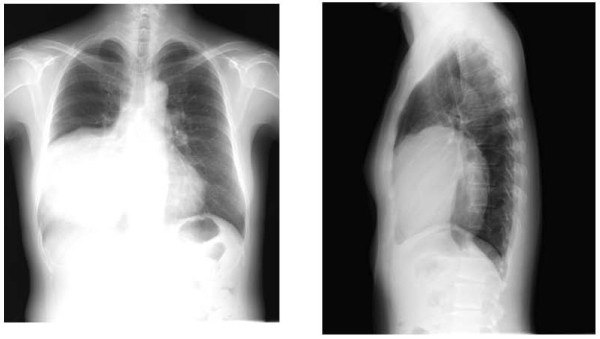
**Plain chest radiograph showing a mass lesion in the right lower lung**.

**Figure 2:**
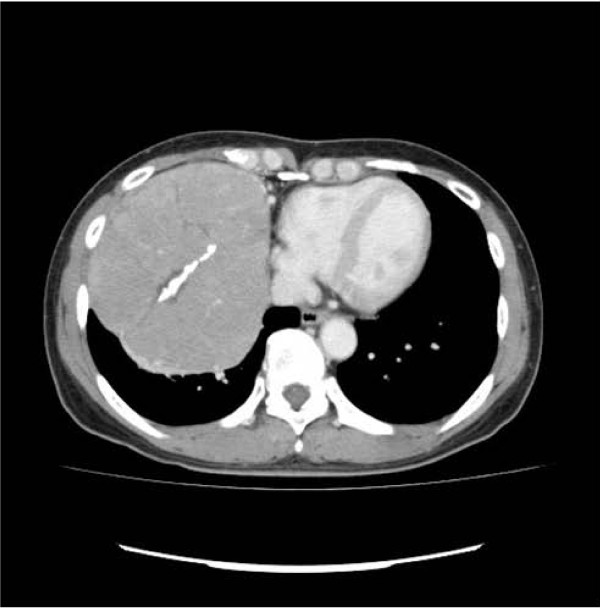
**Chest computed tomograph showing a solid tumor (15 × 10 × 8 cm) with a clear borders and internal calcification in the right thoracic cavity**.

**Figure 3:**
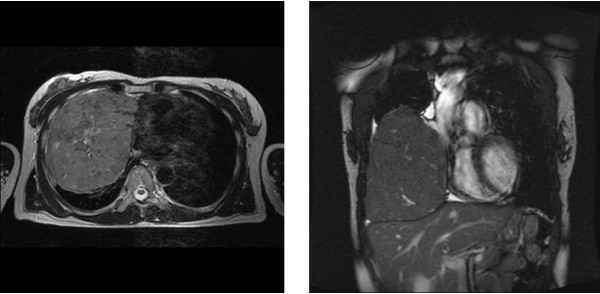
**Chest magnetic resonance imaging**. The tumor appeared isointense relative to the skeletal muscle on T1-weighted image, while T2-weighted images show partial inclusion of weak signal hypointensities of moderate signal strength.

**Figure 4:**
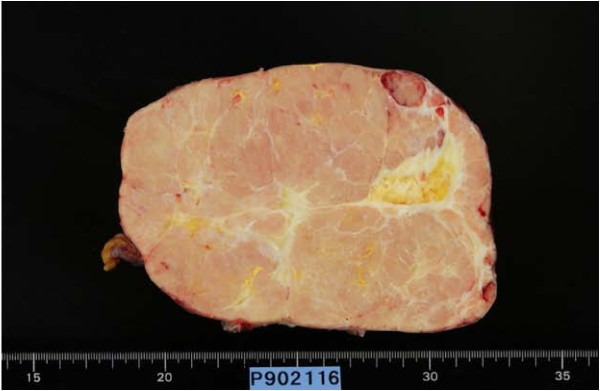
**Excised specimen of the tumor showing a smooth margin and calcification within**.

**Figure 5:**
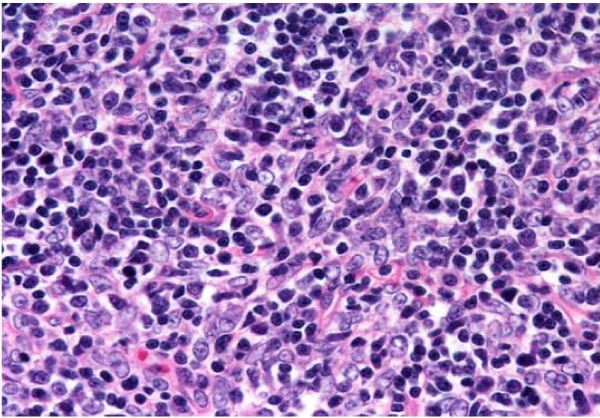
**Histopathological examination (hematoxylin and eosin, × 400) revealed abundant lymphocytes and large, bright tumor cells**. Cells with chromatin-poor nuclei are evident.

**Figure 6:**
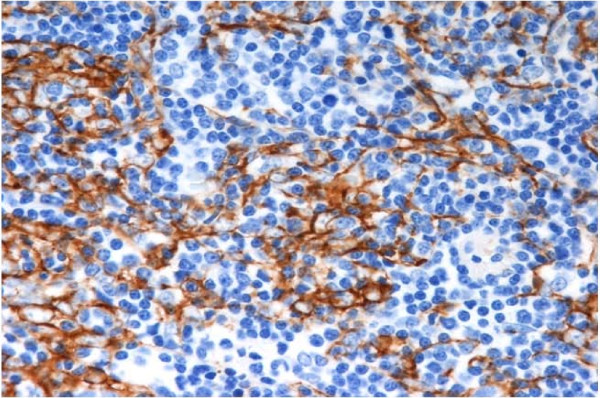
**Immunohistochemical staining (keratin staining × 400) showing epithelial cells distributed in a mesh-like form, mixed among lymphocytes at a ratio of nearly 1:1**.

A 50-year-old Japanese woman with the chief complaint of chest pain was examined at a local hospital. A chest radiograph revealed a giant tumor in the right lower lung field, and the patient was referred to our department. The patient had no pertinent personal or family history, and had never smoked. Respiratory sounds in the right lower lung field were diminished, but no other abnormalities were detected on physical examination. Respiratory function tests revealed a vital capacity (VC) of 1,470 mL and a percent predicted VC of 46.7%, indicative of restrictive pulmonary disease. No abnormalities were identified on blood biochemistry. A plaine chest x-ray (Figure 1) showed a giant tumor shadow measuring 15 × 13 cm in the right lower lung field, and chest computed tomography (Figure 2) showed a solid tumor measuring 15 × 10 × 8 cm in the right thoracic cavity. The tumor showed a clear borders and internal calcification, and was found todisplace the diaphragm downward and the heart to the left. In addition, on chest magnetic resonance imaging (MRI) (Figure 3), the tumor was visualized as an isointensity relative to the skeletal muscle on T1-weighted images, while on T2-weighted images, partial inclusion of weak signals hypointensity of moderate signal strength. Moreover, diffusion-weighted imaging revealed slightly heterogeneous signal hyperintensity, but no findings suggestive of degeneration or necrosis. A fibrous septum was found within the tumor, which showed a trabecular growth pattern, which led to the diagnosis of SFT, and surgery was performed. A small, the sixth intercostal video-assisted thoracotomy was performed, and the tumor was found to be slightly adherent to the diaphragm, mediastinal pleura, and lower lobe of the right lung. However, the tumor could be relatively easily dissected from these organs and removed. No continuity with normal thymic tissue was seen. Intraoperative rapid pathological diagnosis indicated a thymoma or lymphoma, and the surgery was terminated. The excised specimen (Figure 4) showed a tumor measuring 5 × 10 × 8 cm and weighing 430 g, covered by a thin, fibrous membrane. The mass was elastic and soft, and the cut surface was lobulated and pale brown in color. Histopathological examination of sections stained with hematoxylin-eosin (HE) (Figure 5) revealed abundant lymphocytes and large, bright tumor cells. No invasion of the capsule was evident, leading to the diagnosis of a Masaoka stage I thymoma. Immunohistochemical staining (Figure 6) showed mature lymphocytes mainly composed of T cells, mixed in a complex pattern with cytokeratin-positive epithelial cells. These findings led to the diagnosis of lymphocyte-predominant thymoma (type B1 thymoma). The postoperative course was good, and the patient has shown no evidence of recurrence as at the time of writing.

## Discussion

Thymomas are tumors developing mainly in the thymus, are located in the anterior mediastinum, with 96% of the tumors occurring in the anterior or anterosuperior mediastinum, and only 4% being ectopic tumors [1,2]. Ectopic thymomas have been described in the neck [3], middle mediastinum [4,5] posterior mediastinum, lung [6], and pleura [7,8], few reports have described giant intrathoracic tumors. Thymomas are generally asymptomatic, but symptoms such as chest pain and respiratory discomfort can be caused by compression of the surrounding organs due to growth of the tumor. Symptoms such as superior vena cava syndrome can also be coused by tumor invasion of the surrounding tissues, myasthenia gravis, pure red cell apalasia, hypogammaglobulinemia. These symptoms/complications can lead to the discovery of the tumor. In addition, some patients show multiple lung metastases or pleural dissemination arising from recurrence or metastasis. The intrathoracic tumor in the present patient was discovered during the course of a clinical workup for chest pain, caused by compression of the surrounding organs. The patient also showed restricted impairment of pulmonary function due to the pressure on normal lung tissue, and surgical removal of the thymoma as quickly as possible was therefore considered necessary.

Definitive diagnosis is needed before surgical removal of a thymoma is planned. The differential diagnosed for giant intrathoracic tumors include SFTs, tumors of pleural origin, such as malignant pleural mesothelioma or sarcoma, chest wall tumors, and metastatic tumors. In our patient, SFT was initially suspected on the basis of the MRI findings, including the shape, signal status under various weightings, and the presence of numerous linear non-signals that were considered to indicate flow voids within the lesion. It was considered that percutaneous needle biopsy would yield a definitive diagnosis, but this procedure was not performed considering the risk of tumor cell dissemination and bleeding from the tumor. Hemorrhagic shock induced by spontaneous rupture of a giant thymoma has been reported [9], and caution is warranted when considering biopsy.

## Consent statement

Informed consent was obtained from the patient for publication of this case report and of the accompanying images. A copy of the written consent is available for review by the Editor-in-Chief of this journal.

## Competing interests

The authors declare that they have no competing interests.

## Authors' contributions

MK operated on this case and analyzed all the data. KO, KS, YM, and SH assisted in the operation. YT and NM diagnosed the pathology of this case. TS did a professor of the Department of Surgery and had the guide of this paper. All authors read and approved the final manuscript.
